# P-672. Coinfections with Respiratory Syncytial Virus (RSV) in a Cohort of Adults with Pre-Existing Comorbidities in Wisconsin from 2015-16 through 2019-20

**DOI:** 10.1093/ofid/ofae631.868

**Published:** 2025-01-29

**Authors:** Oluwakemi Alonge, Maria Sundaram, Huong Nguyen, Jennifer P King, Elisha Stefanski, Pouya Saeedi, Yves Brabant, Jean-Yves Pirçon

**Affiliations:** Marshfield Clinic Research Institute, Marshfield, Wisconsin; Marshfield Clinic Research Institute, Marshfield, Wisconsin; Marshfield Clinic Research Institute, Marshfield, Wisconsin; Marshfield Clinic Research Institute, Marshfield, Wisconsin; Marshfield Clinic Research Institute, Marshfield, Wisconsin; GSK, Wavre, Brabant Wallon, Belgium; GSK, Wavre, Brabant Wallon, Belgium; GSK, Wavre, Brabant Wallon, Belgium

## Abstract

**Background:**

Current evidence suggests that coinfections with respiratory syncytial virus (RSV) may affect symptom and disease severity during acute respiratory infection (ARI). However, this data is largely observed in children, with limited data regarding RSV coinfections in adults with pre-existing comorbidities.

**Table 1.**
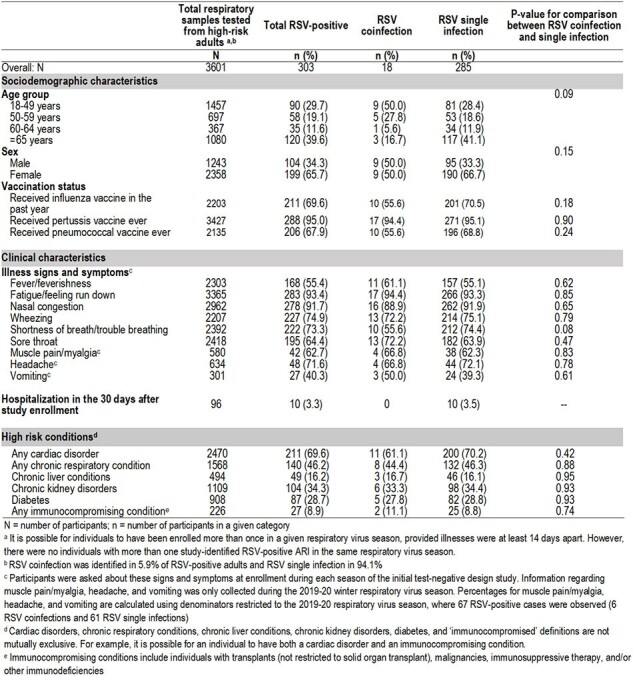
Demographic and clinical characteristics among high-risk adults with RSV coinfection and RSV single infection from 2015-16 through 2019-20 respiratory virus seasons.

**Methods:**

We conducted a retrospective cohort study, using existing data and respiratory specimens from community-dwelling adult participants in a test-negative study of influenza vaccine effectiveness. The study was conducted in an outpatient setting in Wisconsin from 2015-2016 through 2019-2020. Participants included in this analysis had ≥1 pre-existing comorbidity (Table 1). Residual respiratory specimens were retested using a PCR-based multiplex panel for adenovirus (HAdV), seasonal coronaviruses (HCoV), human metapneumovirus, rhinovirus/enterovirus (HRV), influenza virus (IFV) (influenza A H1pdm09, H3, and B), human parainfluenza virus types 1-4, RSV A and B. RSV coinfection was defined as the detection of two or more pathogens, one of which was RSV A or B and the other non-RSV. We used chi-square tests to compare demographic and clinical characteristics between those with RSV coinfection vs. RSV single infection.

**Results:**

Of 3601 respiratory samples from high-risk adults tested, RSV coinfection was detected in 18 (0.5%) participants and RSV single infection in 285 (7.9%). RSV coinfections occurred with HRV (8; 44.4%); IFV (6; 33.3%); HCoV (5; 27.8%); and HAdV (1; 5.6%), and was highest among those aged 18-49 years (9; 50%). We did not observe statistically significant differences between coinfections vs. single infections by presence of upper or lower respiratory symptoms, or by high-risk conditions (Table 1). Hospitalization occurred only in individuals with RSV single infections (10, 3.5%).

**Conclusion:**

RSV viral co-infections were detected in 0.5% of 3601 respiratory samples of high-risk adults, including coinfections with HRV, IFV, HCoV and HAdV. We did not observe significant differences in symptoms between those with RSV coinfection vs single infection, however, hospitalization occurred only in individuals with RSV single infection. Limitations of this analysis include its sample size and the restrictions of multiplex assay sensitivity and specificity.

**Disclosures:**

**Oluwakemi Alonge, MPH, CPH**, CSL Seqirus: Grant/Research Support|GSK Inc.: Grant/Research Support **Maria Sundaram, PhD, MSPH**, GSK Inc.: Grant/Research Support|Pfizer Inc.: Grant/Research Support **Huong Nguyen, PhD, MPH**, CSL Seqirus: Advisor/Consultant|CSL Seqirus: Grant/Research Support|GSK: Grant/Research Support|ModernaTX: Advisor/Consultant|ModernaTX: Grant/Research Support **Jennifer P. King, MPH**, GSK Inc.: Grant/Research Support|ModernaTX: Grant/Research Support **Elisha Stefanski, BS**, CSL Seqirus: Grant/Research Support|GSK Inc.: Grant/Research Support|ModernaTX: Grant/Research Support **Pouya Saeedi, PhD.**, GSK: Employment **Yves Brabant, MEng**, GSK: Employed **Jean-Yves Pirçon, PhD**, GSK Inc.: Employment

